# Sexual Assault: Approach to Reality in the Area of Santiago de Compostela (Galicia, Spain) through a 12-Year Retrospective Study

**DOI:** 10.1093/jat/bkac080

**Published:** 2022-10-07

**Authors:** P Cabarcos-Fernández, M J Tabernero-Duque, I Álvarez-Freire, A M Bermejo-Barrera

**Affiliations:** Forensic Toxicology Service, Institute of Forensic Sciences, Faculty of Medicine, University of Santiago de Compostela, C/San Francisco s/n, Santiago de Compostela 15782, Spain; Forensic Toxicology Service, Institute of Forensic Sciences, Faculty of Medicine, University of Santiago de Compostela, C/San Francisco s/n, Santiago de Compostela 15782, Spain; Forensic Toxicology Service, Institute of Forensic Sciences, Faculty of Medicine, University of Santiago de Compostela, C/San Francisco s/n, Santiago de Compostela 15782, Spain; Forensic Toxicology Service, Institute of Forensic Sciences, Faculty of Medicine, University of Santiago de Compostela, C/San Francisco s/n, Santiago de Compostela 15782, Spain

## Abstract

Sexual assault constitutes a severely traumatic experience that impacts the lives of far too many victims each year. The underlying behaviors of the offenders are often associated with psychological, physical and social distress, and the use of psychotropic substances was found in a good number of cases. A descriptive and retrospective review of sexual assault cases has been undertaken to identify trends in the toxicology findings in Drug-Facilitated Sexual Assault in Santiago de Compostela over the past 12 years. During this period, a total of 69 cases were referred to the Forensic Toxicology Service as sexual assault cases. The sex and age distribution of the cases showed that females between the ages of 14 and 65 years constituted the group most frequently submitted to sexual assault, with a peak of 55.1% in the 18- to 30-year age group. Alcohol consumption was positive in 77.1% of positive cases determined, followed by drugs (34.4%) and illicit drugs (26.2%). Our results showed a high percentage of alcohol consumption in sexual assault cases.

## Introduction

Drug-facilitated crime includes rape or other sexual assault, robbery, money extortion, as well as the deliberate maltreatment of vulnerable people under the manipulation of psychotropic substances (1). The intake of psychoactive substances either voluntarily or not, leading to episodes of sexual assault has become a relevant social issue account made of its well-documented health consequences (2–5). The term Drug-Facilitated Sexual Assault (DFSA) comprises a subset of sexual violence cases (6–8) where non-consenting sexual contact is facilitated by drug use. The most common substances triggering DFSA episodes are, according to a literature search, ethanol, benzodiazepines, gamma-hydroxybutyrate (GHB), ketamine (3, 9–11) and, to a lesser extent, analgesics, antidepressants, antihistamines, some antipsychotics and barbiturates.

The incidence of sexual assault cases is known to be underreported, as well as the type and prevalence of the drug used, as there are several factors involved, such as the victim’s embarrassment in reporting abuse, the time elapsed between the fact and the complaint and the lack of adequate instrumentation for detection, since the most used drugs for this purpose have a short half-life and are quickly metabolized and eliminated from the victim’s body without leaving a trace (3). In addition, the fact that most of the published cases of sexual assaults involve a significantly high percentage of female cases reinforces the hypothesis that sexual assaults among men are largely underreported (1, 12).

The toxicological analysis these drugs can be done in several biological matrices, such as urine, plasma, whole blood, oral fluid and hair. In general, blood samples are the most widely used specimens for drug detection, although it has a rather reduced time window of detection, comprising only 24–48 h after administration (3).

Many studies on sexual assaults appear in scientific bibliography (1, 2, 5, 11, 13–17). However, there are currently no published studies on the toxicological aspects in relation to sexual assaults in Galicia.

This study involves those cases analyzed at the Forensic Science Institute of the University of Santiago de Compostela and sent to our facilities by Instituto de Medicina Legal de Galicia (Institute of Legal Medicine of Galicia; IMELGA). This organization integrates Galicia’s coroners, distributed in the main Galician cities. The purpose of this study is to make a descriptive and retrospective review of sexual assaults in Santiago de Compostela, the capital of the Galicia region in northwestern Spain with a population of around 184,848 inhabitants, from 2009 to 2021.

## Materials and Methods

The present work represents a descriptive and retrospective study of data collected between 2009 and 2021 in the Forensic Toxicology Service, University of Santiago de Compostela. All our cases reported as sexual assaults were considered.

Forensic medical examinations are performed to collect evidence to aid prosecutions. Biological samples were previously collected by coroners and sent to our Service for analysis. Normally, biological samples received involve blood, urine and/or hair. All samples were analyzed for the presence and concentration of alcohol, drugs and illicit psychoactive substances using the appropriate detection techniques. Enzyme immunoassay techniques were used to determine cocaine, opiates, amphetamines, benzodiazepines, methadone, cannabis and ethyl glucuronide (EtG) in urine whenever possible. All these substances and those not determined by enzyme immunoassay techniques, such as GHB, antidepressants or anesthetics, were determined and confirmed by gas chromatography–mass spectrometry and/or high-performance liquid chromatograph coupled to a photodiode-array detector. The presence of alcohol was determined using a gas chromatograph–flame ionization detector. The most relevant information about these cases, which includes age, biological samples employed and toxicological results, is shown in Table I.

**Table I.** Information about Sexual Assault Victims from Santiago de Compostela Received at Forensic Toxicology Service

Case no	Age	Ethanol (blood)	Ethanol (urine)	EtG	Other samples	Other substances
1	21	NA	Negative	NA		
2	27	Positive	Positive			
3	17	Positive	NA			
4	28	Positive	NA			
5	17	Positive	Positive			
6	21	Negative	Negative	NA		
7	19	Positive	Positive			Cocaine, cannabis, propofol, Bzd, Metamizole
8	18	Positive	NA			
9	17	Positive	NA			
10	23	NA	Positive			Cocaine, cannabis, Bzd
11	32	Positive	Positive			
12	38	Negative	Negative	Positive		
13	24	Negative	Negative	Positive		MDMA, cocaine
14	19	Negative	Negative	Positive		
15	25	NA	Negative	NA		Bzd
16	24	Positive	NA			
17	18	Negative	NA	NA		
18	15	Positive	Positive			
19	24	Positive	NA			
20	30	Positive	Positive			
21	31	NA	Negative	Positive	Hair*	Cocaine; Cannabis; MDMA
22	36	Negative	Negative	Positive	Hair*	MDMA; Cocaine; Cannabis
23	18	Negative	Negative	Positive		
24	65	Negative	Negative	Negative		Pseudoephedrine; Triprolidine; Bzd; Cetirizine; Metamizole
25	44	Negative	Negative	Negative		Bzd; Venlafaxine
26	19	NA	Positive			Clomethiazole
27	19	NA	Positive			Clomethiazole
28	27	Positive	Positive			
29	19	Positive	Positive			
30	20	Positive	NA			
31	37	Negative	Negative	Negative		Cocaine
32	46	Positive	Positive			Cocaine; Methadone; Topiramate
33	15	NA	NA		Hair	Cocaine; Cannabis
34	32	NA	NA		Hair	
35	43	NA	Negative	NA		Topiramate; Carbamazepine
36	21	Negative	Negative	Positive		
37	50	Positive	Positive			
38	32	NA	NA		Hair	
39	18	Negative	Negative	Positive		
40	42	NA	Negative	Negative		
41	37	Negative	Negative	Negative		Bzd
42	55	NA	Negative	Negative		Bzd, Metamizole
43	50	Negative	Negative	Negative		
44	16	Positive	Positive			
45	19	Positive	Positive			
46	14	NA	Negative	NA		Bzd, cannabis
47	27	Positive	Positive			Cocaine
48	16	NA	Positive			
49	18	Positive	Positive			
50	55	NA	Negative	Negative		Bzd; Cannabis
51	19	Negative	Negative	Negative		MDMA, MDA
52	20	Positive	Positive			Paracetamol; Ibuprofen
53	32	NA	Negative	Negative		
54	29	Positive	Positive			
55	15	Positive	Negative			
56	40	Negative	Negative	Positive		Cocaine; Cannabis; Citalopram
57	24	Negative	Negative	Negative		Cocaine
58	21	Positive	Positive			
59	16	NA	Negative	Positive		Lamotrigine; Ibuprofen
60	29	Negative	NA	Negative		Zolpidem; Sertraline
61	21	NA	Positive			Cocaine; Methadone; Mirtazapine; Metamizole; Opiates; Cannabis
62	19	Positive	Positive			
63	17	Positive	Positive			
64	20	Positive	Positive			Bzd
65	41	Negative	Negative	Positive		Bzd, Carbamazepine, Venlafaxine
66	26	Negative	Negative	Positive		
67	20	Positive	Positive		Hair*	
68	23	Positive	Positive		Hair*	
69	23	Negative	Negative	Negative		Cocaine; Cannabis; Paracetamol; Venlafaxine

EtG: ethyl glucuronide; Bzd: benzodiazepines; NA: not available.

* Hair: hair sample collected 2 months after the sexual assault.

## Results

A total of 69 cases were received between 2009 and 2021. These relatively low number of assaults correspond to a small area of Spain. The data, however, reveals a slight upward trend. It is also necessary to mention the slight decrease in the number of cases during 2020, the first year affected by COVID-19. The number of cases received in our laboratory has been significantly affected by the current global pandemic. Table I shows information about these cases received in our service.

According to the current data received in our laboratory as cases of sexual assault, 2 males and 67 females aged between 14 and 65 years were registered as victims of sexual assault from 2009 to 2021 in Santiago de Compostela. When analyzed by age group, 55.1% of the victims were in the 18- to 30-year age group.

Biological samples from victims of sexual assault were sent to Forensic Toxicology Service to perform the analysis. The samples received have been mainly blood, urine and/or hair.

Toxicological analysis was positive for some substances in 88.4% of the total cases. Of the 61 cases with positive results for some substances, 77.1% of them were positive for alcohol (52.5% of the positive cases presented only alcohol consumption), 34.4% for drugs (mainly benzodiazepines) and 26.2% for illicit drugs (mainly cocaine followed by cannabis), alone or in combination with others (Table II; Supplementary Figure 1SM). These results are similar to those obtained by García-Caballero et al. (18).

**Table II.** Substances Found in Sexual Assault Cases (Alone or in Combination with Others)

Substance	Frequency	% of the positive cases	Female♀	Male♂
Alcohol	47	77.1% (52.5% only alcohol consumption)	46	1
Drugs	21	34.4%	20	1
Illicit drugs	16	26.2%	14	2

Of the 69 cases received, we found 35 positive cases for ethanol, 31 negative and 3 cases where it was not been possible to determine since only hair samples were made available. Of the 31 negative cases, the EtG determination was applied to 25 urine cases, determining 12 new positive alcohol cases. There were six cases in which the EtG determination could not be performed because at the moment these cases were received in our laboratory, we did not have the specific technique available.

Voluntary alcohol ingestion was reported in 13% of subjects (9 cases over the age of 15 years), while this information was not available for 60 cases. Cannabis use was reported less frequently, by only 7.2% of the victims.

## Discussion

Sexual assault is a common form of trauma. According to Dworkin et al., 17–25% of women and 1–3% of men are sexually assaulted in their lifetime (19, 20). This means that it should be considered as an international public health problem.

This work reports on a 12-year retrospective study of cases of sexual assault in Santiago de Compostela. The results yield a low incidence rate when compared to other communities within Spain, probably because Galicia is one of the autonomous communities with the lowest and older population.

Sexual assault encompasses both genders and all ages, including children and the elderly.

In this study, there is a clear predominance of female victims (*N* = 67; 97.1%) versus males (*N* = 2; 2.9%). Few data are available on sexual assaults perpetrated by women. Duchesne et al. reported two possibilities: on the one hand, few men report sexual assault perpetrated by women because of the strong negative impact of the aggression and, on the other hand, because the impact is so weak that victims do not feel the need to report it to the police or to a doctor (1).

The age range included in this work was from 14 to 65 years, with the age group of 18 to 30 years having the highest incidence, data that coincides with other reports. (21, 22). Experiencing sexual violence at an early age is associated with numerous adverse health (alcohol use disorders and sexual revictimization), psychological (depression, suicidal tendencies, anxiety, personality disorders and posttraumatic stress disorder) and legal (delinquency and conduct disorder) consequences (11).

The collection of biological matrices is also essential in DFSA cases. Blood and urine were the most employed samples involved in the determination of the cases. Blood testing can be useful in gaining more insight into the timeframe of substance exposure in relation to the crime. However, it usually takes a while for the sexual assault victim to report. In these cases, urine is a useful sample due to its wider window of detection. On the contrary, the use of hair for this type of cases requires extremely sensitive techniques for the detection of the possible substances involved, since, in general, it is a single-dose intake. In addition, the time required for the incorporation of drugs into this matrix needs to be taken into account.

The time elapsed between the sexual assault and the sampling processing usually varies depending on the reaction time of each victim. In most of our cases, the victims have gone to a medical center some 12–72 hours after the events. In 3 of the 7 cases in which hair samples were received, the sample was taken at the same time as the blood and urine. In the remaining 4, the sample was collected 2 months later.

Of the 69 cases we received as possible sexual assault, 61 positive cases (88.4%) have been determined for any of the substances considered in this study (alcohol, main drugs of abuse and drugs). Table I gives a list of substances encountered in this study.

In our study, more than half of the positive cases were only for alcohol (52.5%). The fact that experiencing sexual assault is associated with alcohol use is consistent with most of the literature (1–3, 10–12, 14). Most of the time it is a voluntary intake (23). In fact, some studies show that reducing excessive alcohol consumption can mitigate sexual aggression since its intake creates a situation that could trigger aggression (9–11, 24). McMullin and White also found that victims of sexual assault report more alcohol consumption than non-victims (25). However, according to Norris et al., the association between heavy drinking and the risk of sexual assault is far from definitive (13).

The analysis of ethanol in samples collected a long time after the incident could generate false-negative results. In these cases, the analysis of its metabolite EtG is recommended (3). EtG is eliminated much more slowly than ethanol, which extends the detection time window; EtG is present in urine for a longer time (80 hours) than ethanol (≈24 hours). Therefore, in the case of sexual assault, in which the time between the alleged crime and the sampling time is more than 20 hours, it is recommended to determine the EtG and to use urine as the biological matrix. In this study, it was possible to increase a 19.6% positivity to alcohol consumption by determining the EtG.

On the other hand, the illicit drug and the family of drugs most detected in this study were cocaine and benzodiazepines, respectively, followed by the cannabis and analgesics/antipyretics group. However, the interpretation of positive results for some substances, such as tetrahydrocannabinol (THC) or benzodiazepines, must be done carefully as they have a long elimination half-life, and a positive finding might or might not be related to the offense. Nevertheless, the psychoactive potential of some of them, like cannabis, must not be underestimated and its synergy of effects with alcohol and other central nervous system depressants should be kept in mind when interpreting cases of DFSA.

The results of this study showed that, after alcohol, pharmaceuticals rather than illicit drugs are substances mostly involved, especially benzodiazepines. This fact is in agreement with the scientific bibliography (9, 26–33).

Despite the lack of data on cases of sexual assault received in our laboratory, the results are alarming as they reflect a significant consumption of ethyl alcohol and/or other illicit substances or drugs in this type of crime.

The current outlook that emerges from an extensive range of publications in terms of DFSA in Spain is similar to the situation present in neighboring countries. It is estimated that about 17% of sexual assaults could be considered as DFSA cases (6). However, due to the limited information made available to us, it has not been possible in many of the cases to distinguish whether or not drug intake did involve voluntary consumption by the victim.

## Conclusions

The high prevalence of sexual assault is particularly concerning in light of its significant psychological consequences, especially on mental health (1, 19). The difficulty in detecting cases of DFSA is well known, so the work of the toxicology laboratory is very important since it allows to demonstrate if the victim was under the effects of some substances that could override her/his will. Our data reveals alcohol as the most employed substance followed by prescription drugs and, finally, illicit drugs.

A good knowledge of the case and collaboration with the coroners are essential to handle this type of cases effectively.

### Study limitations

Interpretation of the present findings should consider certain limitations already mentioned in the text. On the one hand, a limitation of our study is that we do not have information on all cases of sexual assault in Galicia. On the other hand, the number of male victims in our study is low, as reported by the works present in the scientific bibliography, since men do not usually file a complaint.

## Supplementary Material

bkac080_Supp

## Data Availability

Data of a sensitive nature have not been published due to the data protection law. All non-sensitive information is included in the article.
