# Supplementary figures and images for: Sexual Assault: Approach to Reality in the Area of Santiago de Compostela (Galicia, Spain) through a 12-Year Retrospective Study
Source: J Anal Toxicol. 2022 Oct 7;46(9):e218–22. doi: 10.1093/jat/bkac080 (PMC9872222; doi:10.1093/jat/bkac080)

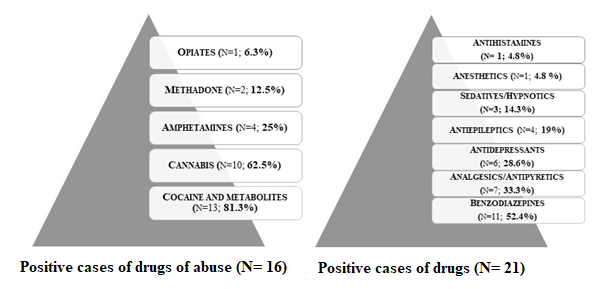

Supplement: bkac080_Supp [file bkac080_supp.zip › jat-22-3713-File002.tiff]
