# Supplementary material for: Sexual Assault: Approach to Reality in the Area of Santiago de Compostela (Galicia, Spain) through a 12-Year Retrospective Study
Source: J Anal Toxicol. 2022 Oct 7;46(9):e218–22. doi: 10.1093/jat/bkac080 (PMC9872222; doi:10.1093/jat/bkac080)
Supplement: bkac080_Supp [file bkac080_supp.zip › jat-22-3713-File003.doc]

| Figure 1SM | Number of positive cases for drugs of abuse (and main drugs of abuse found; N= 16). Number of positive cases for drugs (and main drugs found: N= 21) |
| --- | --- |
